# *Epimedium* Aqueous Extract Ameliorates Cerebral Ischemia/Reperfusion Injury through Inhibiting ROS/NLRP3-Mediated Pyroptosis

**DOI:** 10.3390/antiox12050999

**Published:** 2023-04-25

**Authors:** Xiaoyu Wu, Jiajia Wei, Yang Yi, Guotao Shu, Zhixu He, Qihai Gong, Jianmei Gao

**Affiliations:** 1 Key Laboratory of Basic Pharmacology, Ministry of Education and Joint International Research Laboratory of Ethnomedicine, Zunyi Medical University, Zunyi 563000, China; 2 Key Laboratory of Basic Pharmacology of Guizhou Province, Department of Pharmacology, School of Pharmacy, Zunyi Medical University, Zunyi 563000, China; 3 The Collaborative Innovation Center of Tissue Damage Repair and Regeneration, Medicine of Zunyi Medical University, Zunyi 563000, China

**Keywords:** *Epimedium*, cerebral ischemic/reperfusion injury, neuron loss, neuroinflammation, NLRP3

## Abstract

Cerebral ischemia/reperfusion causes exacerbated neuronal damage involving excessive neuroinflammation and oxidative stress. ROS is considered a signal molecule to activate NLRP3; thus, the ROS/NLRP3/pyroptosis axis plays a vital role in the pathogenesis of cerebral ischemia/reperfusion injury (CIRI). Therefore, targeting the inhibition of the ROS/NLRP3/pyroptosis axis may be a promising therapeutic tactic for CIRI. *Epimedium* (EP) contains many active ingredients (ICA, ICS II, and ICT), which have a wide range of pharmacological activities. However, whether EP can protect against CIRI remains unknown. Thus, in this study, we designed to investigate the effect and possible underlying mechanism of EP on CIRI. The results showed that treatment with EP dramatically mitigated brain damage in rats following CIRI, which was achieved by suppressing mitochondrial oxidative stress and neuroinflammation. Furthermore, we identified the ROS/NLRP3/pyroptosis axis as a vital process and NLRP3 as a vital target in EP-mediated protection. Most interestingly, the main compounds of EP directly bonded with NLRP3, as reflected by molecular docking, which indicated that NLRP3 might be a promising therapeutic target for EP-elicited cerebral protection. In conclusion, our findings illustrate that ICS II protects against neuron loss and neuroinflammation after CIRI by inhibiting ROS/NLRP3-mediated pyroptosis.

## 1. Introduction

Ischemic strokes are a leading cause of mortality and long-term adult disability [1]. Ischemia generates irreversible damage, such as neuronal death, in the cerebral ischemic core because of a deficiency in blood supply. However, timely reperfusion restores neuronal survival in the penumbra area [2]. Currently, the recombinant tissue-type plasminogen activator (rtPA) for brain recanalization aggravates ischemic injuries and results in cerebral ischemic/reperfusion injury (CIRI) [3]. Therefore, it is of great significance to develop efficacious tactics or therapies for rescuing dying neurons in ischemic penumbra during CIRI.

Compelling evidence suggests that apart from neurons, following CIRI, microglia and astrocytes are activated and then elicit excessive inflammatory responses, known as “cytokine storms”, with the promotion of interleukin (IL)-6, IL-1β, and tumor necrosis factor (TNF)-α release [4]. Cytokine storms cause pernicious neuroinflammation on the salvation and recovery of cerebral ischemia, along with the generation of oxidative stress and pyroptosis. Oxidative stress, a disorder in the equilibrium between the generation of reactive oxygen species (ROS, e.g., hydrogen peroxide, superoxide) and antioxidant defenses systems, is elicited during CIRI peculiarly through neuroinflammation, which promotes the generation of excessive reactive oxygen species (ROS) [5]. Pyroptosis is a novel programmed cell death process modulated by Gasdermins (GSDMs) and is also deemed an inflammatory cell death associated with neuroinflammation. Pyroptosis is initiated by the activation of NOD-like receptor 3 (NLRP3), apoptosis-associated speck-like protein containing a caspase activation and recruitment domain (ASC), caspase-1, and GSDMD-N, which controls the cleaving and activation of IL-18 and IL-1β [6]. Recent evidence demonstrates that ROS are considered signal molecules that activate NLRP3, and the ROS/NLRP3/pyroptosis axis plays a vital role in the pathogenesis of CIRI [7]. Thus, targeting the inhibition of the ROS/NLRP3/pyroptosis axis might be a promising therapeutic tactic for CIRI.

**Figure 1:**
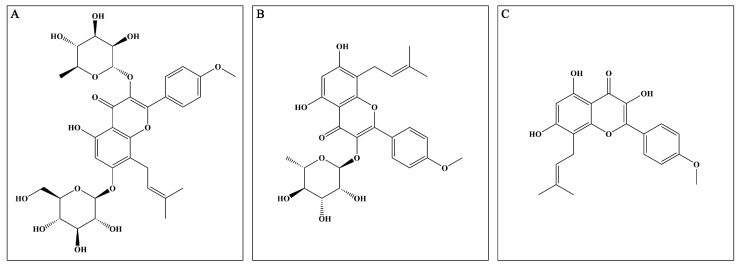
Major active compounds (flavonoids) found in EP. (**A**) ICA; (**B**) ICS II; (**C**) ICT.

*Epimedium* (EP), derived from the *Epimedium* genus, has been utilized alone and in combination with other Chinese herbal medicines to treat multiple disorders, such as osteoporosis, erectile dysfunction, and cardiovascular diseases [8]. Mounting reports prove that EP possesses excellent anti-inflammatory and antioxidant properties, which indicate that it may exert potential neuroprotective effects. The major active compounds (flavonoids) present in the dried aerial parts of EP are icariin (ICA), icariside II (ICS II), and icaritin (ICT) (Figure 1). However, up to now, the mechanism of EP on CIRI remains unclear. Therefore, the possible underlying mechanism of EP against CIRI in an animal model was comprehensively and systematically investigated in the present study.

## 2. Materials and Methods

### 2.1. Preparation and Analysis of EP

The effective components of EP were extracted by decoction in distilled water twice (0.1 g/mL; 1.5 h each time) and then combined [9]. The combined extracts were cooled to room temperature, evaporated, and concentrated using a rotary evaporator, next made into a solid block using a vacuum freeze dryer, then ground into powder and stored at −20 °C in a dry, sealed and non-polluting condition until the next use. LC-MS/MS analysis was performed for quality control of the EP extracts utilizing Phenomenex Gemini C18 combined with a 4000 QTRAP mass spectrometer (SCIEX) at a column temperature of 25 °C. The mobile phase (A: 0.1% formic acid in acetonitrile; B: an aqueous solution containing 0.1% formic acid) was delivered at a speed of 0.35 mL/min: 0–6 min (35–55%A); 6–9.5 min (55–100%A); and 9.5–15 min (100%A).

### 2.2. Animals

Animal experiments were carried out in strict compliance with the Guidelines for the Care and Use of Laboratory Animals of the National Institutes of Health (NIH Publication No. 8023, revised 1978) and were approved by the Ethics Committee of Zunyi Medical University (Guizhou, China). Specific pathogen free (SPF) male Sprague Dawley (SD) rats (7–8 weeks, 260–280 g) were purchased from Hunan SJA Laboratory Animal Co., Ltd. (Certificate number: SCXK (Xiang) 2019-0004, Changsha, China). The rats were housed five to six per cage in the SPF class animal house in the Key Laboratory of Basic Pharmacology of the Ministry of Education, Zunyi Medical University, with free access to a standard rodent diet and water. The room temperature was maintained at about 25 ± 2 °C and the humidity at 50% ± 5%, while the alternating light and dark cycles were maintained at 12 h/12 h.

### 2.3. Model Preparation

The focal cerebral ischemia model was induced by intraluminal occlusion of the right middle cerebral artery (MCA), as described in a previous study [10]. In short, rats were fasted for 24 h and given intraperitoneal sodium pentobarbital anesthesia (50 mg/kg). Then, a 0.36 mm diameter silicon-coated 5/0 monofilament nylon suture was plugged into the internal carotid artery through the external carotid artery stump until slightly resistant to occlude the origin of the middle cerebral artery. After 2 h, reperfusion was established by the withdrawal of the filament.

### 2.4. Laser Speckle Contrast Imaging (LSCI) Monitoring 

The LSCI system (moorO2Flo, Moor Instruments, Millwey Axminster Devon EX13 5HU, Axminster, UK), as described in previous studies, could provide cerebral blood flow (CBF) information with a high spatial and temporal resolution before, during, and immediately after the MCA occlusion model (MCAO) in rats [11]. When the relative cerebral blood flow (rCBF) dropped below 20% and recovered to more than 80% of the baseline, an MCAO model was considered successful. During the imaging procedure, the rat was anesthetized with sodium pentobarbital, and a midline incision was made to fully expose the skull. The LSCI system exposure time was 50 ms, and the frame rate was 20 Hz. Similar imaging sections were recorded at different time points based on the experimental design. 

Subsequently, SD rats were randomly divided into six groups: sham group, sham + EP (7.29 g/kg) group, MCAO group, MCAO + EP (0.81 g/kg) group, MCAO + EP (2.43 g/kg) group, and MCAO + EP (7.29 g/kg) group. The animals were administered EP by gavage at dosages of 0.81, 2.43, and 7.29 g/kg at the start of reperfusion twice a day for three days, whereas rats in the sham and model groups were given volume-matched distilled water instead.

### 2.5. Neurobehavioral Assessment

Neurological deficit scores were evaluated three days after MCAO and were scored on a five-point scale as follows: grade 0, no deficit; grade 1, failure to fully extend left forepaw; grade 2, circling to the left; grade 3, falling to the left; and grade 4, unable to walk spontaneously accompanied with a depressed level of consciousness [12]. In addition, the test was performed by three examiners blinded to the group treatments.

### 2.6. Measurement of Cerebral Infarct Volume

After the neurological test, the infarct size after MCAO was measured using 2, 3, 5-triphenyl-tetrazolium chloride (TTC, #17779, Sigma-Aldrich, Eschenstr.5 82024 Taufkirchen, Germany), as described previously [13]. In brief, brain tissues were separated immediately and frozen at −20 °C for 20 min. Then, five coronal brain slices of 2 mm thickness were dyed with TTC for 60 min in the dark at 37 °C and fixed in 4% paraformaldehyde. Then, the TTC-stained slices were photographed, and the cerebral infarct volume was calculated using imagej.js (v0.5.6; https://ij.imjoy.io/, accessed on 10 April 2022).

### 2.7. Hematoxylin and Eosin Staining (H&E)

After deep anesthetization, the rats went through cardiac perfusion with 200 mL of PBS and then 4% paraformaldehyde. Subsequently, the brains were removed and immersed in 4% paraformaldehyde for 48 h fixation. Then the brain tissue was dehydrated with ethanol and then embedded by using paraffin. Then the embedded brain tissue was cut into sections of 5μm thickness for H&E staining. Concisely, tissue sections were dewaxed with xylene, ethanol, and distilled water. The sections were stained with hematoxylin solution for 5 min, divided with hematoxylin differentiation solution, and then soaked in eosin for 5 min. Dehydration and transparent sealing were then performed for examination under a light microscope (BX 43 Olympus, Tokyo, Japan).

### 2.8. RNA-Seq Analysis and Network Construction

The total RNA of the brain samples (Sham group, MCAO group, MCAO + EP 7.29 g/kg group) was extracted using a Trizol buffer, followed by sample integrity, quality, and purity examinations. Qualified RNA transcriptome was sequenced on a BGISEQ-500RS RNA-Seq platform supported by Beijing Genomics Institute (Shenzhen, China). Gene expression was calculated using the fragments per kilobase of exon model per million mapped fragments (FPKM) of each sample. Principal components analysis (PCA) was used to assess the reliability of the sample. Moreover, gene expression with a fold change (FC) greater than 3 and *p* value less than 0.05 were identified as differentially expressed genes (DEGs) in this study. Evenn (http://www.ehbio.com/test/venn/#/, accessed on 20 September 2022) was applied to draw a Venn diagram. A volcano plot and heatmap were performed using the OmicStudio tools (https://www.omicstudio.cn/tool, accessed on 20 September 2022) [14]. In addition, the DEGs were imported into the STRING 11.5 database (https://string-db.org/, accessed on 20 September 2022), the species was set to Homo sapiens, the screening condition was a combined score ≥ 0.7, and free nodes were deleted. The interaction relationship TSV data file was imported into Cytoscape 3.9.1 software to construct a PPI network, and Cytoscape plugin MCODE was used to screen out the main targets. In addition, ClueGO, a Cytoscape plugin, was used for GO analysis [15]. The R software package clusterProfiler (version 3.14.3) was utilized to analyze the Kyoto Encyclopedia of Genes and Genomes (KEGG) pathway [16].

### 2.9. Molecular Docking Simulations

Molecular docking studies between ICA, ICS II, ICT, and NLRP3 were performed by Autodock 4.2 (RRID:SCR 012746) and Autodock Tools (ADT). The PubChem database was used to collect the 3D molecular structure of ICA, ICS II, and ICT, and the RCSB Protein Data Bank (PDB database, http://www.rcsb.org/, accessed on 10 October 2022) was used to obtain the structure files of the target proteins. Crystal structures of NLRP3 (PDB ID: 6NPY) were employed for molecular docking using PyMOL 2.4 (https://pymol.org/2/, accessed on 10 October 2022) and AutoDock 4.2.

### 2.10. Nissl Staining

The embedded brain tissue was cut into 5 µm thick sections for Nissl staining. The sections were dewaxed and stained with Nissl stain solution (Toluidine Blue Method, G1436, Solarbio, Beijing, China) to detect neuron damage. The densities of neuron and Nissl bodies were quantified with imagej.js (v0.5.6; https://ij.imjoy.io/, accessed on 20 April 2022).

### 2.11. Terminal Deoxynucleotidyl Transferase dUTP Nick End Labeling (TUNEL)

According to the manufacturer, apoptosis in rat samples was detected using the TUNEL kit (C1088, Beyotime), and the results were observed using a fluorescence microscope (BX 53 Olympus, Tokyo, Japan) and imagej.js (v0.5.6; https://ij.imjoy.io/, accessed on 20 November 2022) for statistical analysis.

### 2.12. Immunofluorescence (IF) Staining

Briefly, the procedure for IF staining was as follows: tissue sections were dewaxed with xylene, ethanol, and distilled water, and the sections were further subjected to antigen collection and permeation with 0.3% Triton-X 100, followed by blocking with immunol staining blocking buffer (P0102, Beyotime, Shanghai, China). Then, the sections were incubated with the primary antibodies overnight at 4 °C; after incubation, sections were again incubated with the corresponding secondary antibody. The antibodies used in this manuscript were glial fibrillary acidic protein (GFAP, 1:50, ab7260, Abcam, Cambridge, UK), ionized calcium-binding adaptor molecule 1 (Iba-1, 1:50, ab178847, Abcam), Neuron (NeuN, 1:100, 66836-1-Ig, Proteintech, Wuhan, China), and NLRP3 (1:100, 27458-1-AP, Proteintech). After 4′,6-diamidino-2-phenylindole (DAPI, Sigma-Aldrich) staining, the result was observed by fluorescence microscope (BX 53 Olympus, Tokyo, Japan). 

### 2.13. Enzyme-Linked Immunosorbent Assay (ELISA)

Serum levels of IL-1β (RJ15465, Renjiebio, Shanghai, China), IL-18 (RJ15463, Renjiebio), TNF-α (RJ16622, Renjiebio), IL-6 (RJ15478, Renjiebio), IL-10 (RJ15453, Renjiebio), inducible nitric oxide synthase (iNOS, RJ16565, Renjiebio), malondialdehyde (MDA, RJ15503, Renjiebio), reactive oxygen species (ROS, RJ15780, Renjiebio), catalase (CAT, RJ15737, Renjiebio), superoxide dismutase (SOD, RJ16691, Renjiebio), and glutathione peroxidase (GSH-Px, RJ25745, Renjiebio) were detected using conventional ELISA kits according to the manufacturer’s instructions. In addition, lactate dehydrogenase (LDH) released in rat serum was measured to assess cell damage using the LDH test kit (RJ16172, Renjiebio) according to the manufacturer’s instructions.

### 2.14. Western Blot (WB)

The rats were sacrificed using sodium pentobarbital after three days of treatment with EP, and then the ischemic penumbra was collected as described previously [17]. According to the manufacturer’s instructions, tissue lysate was measured using the BCA protein assay kit (PC0020, Solarbio). Thereafter, the lysates were normalized to equal amounts per group, and 10 μg protein from the tissue lysates was split in sodium dodecyl sulfate-polyacrylamide gel electrophoresis (10%) and transferred to PVDF membrane with 1% blocking buffer (C1090, Beyotime) for 1 h at room temperature. Then, the membranes were incubated with primary antibodies, including NLRP3 (1:1000, ab263899, Abcam), ASC (1:1000, ab17544, Abcam), GSDMD (1:1000, ab219800, Abcam), Cleaved-Caspase 1 (1:1000, #AF4022, Affinity), IL-1β (1:1000, ab254360, Abcam), IL-18 (1:1000, ab191860, Abcam), β-actin (1:5000, ab8227, Abcam), and GAPDH (1:5000, ab8245, Abcam) overnight at 4 °C. Thereafter proteins were determined using the appropriate species-specific HRP-conjugated secondary antibodies for 1 h at room temperature. Representative bands were visualized by ECL WB reagents, and the relative band optical intensity was quantified using imagej.js. The intensity for each protein was normalized to that evaluated for β-actin or GAPDH and was expressed as the relative fold to the sham group.

### 2.15. Materials

The radix of EP was purchased from Yinhua Pharmaceutical Co., Ltd. in Guizhou Province of China. TTC and DAPI were purchased from Sigma-Aldrich (St. Louis, MO, USA). The TUNEL apoptosis assay kit was obtained from Beyotime (Shanghai, China). ELISA kits for IL-1β, IL-18, TNF-α, IL-6, IL-10, iNOS, MDA, ROS, CAT, SOD, GSH-Px, and the LDH test kit were purchased from Shanghai Renjie Bioengineering Institute (Shanghai, China). Antibodies that were utilized against NLRP3, ASC, GSDMD, IL-1β, IL-18, GFAP, Iba-1, β-actin, and GAPDH were purchased from Abcam (Cambridge, UK). Cleaved-Caspase 1 was purchased from Affinity.

### 2.16. Statistics

All values were presented as mean ± standard (mean ± SD) or mean ± standard error of the mean (mean ± SEM) of five or six independent experiments. The number of CFB was compared between groups by Student’s unpaired *t*-test using SPSS statistics 18.0 for Windows (SPSS, Inc., Chicago, IL, USA). In addition, three or more groups were compared using one-way ANOVA followed by Bonferroni (same variance assumed) or Dunnett’s T3 (equal variance not assumed); *p* < 0.05 was considered statistically significant.

## 3. Results

### 3.1. Preparation and Analysis of EP

**Figure 2:**
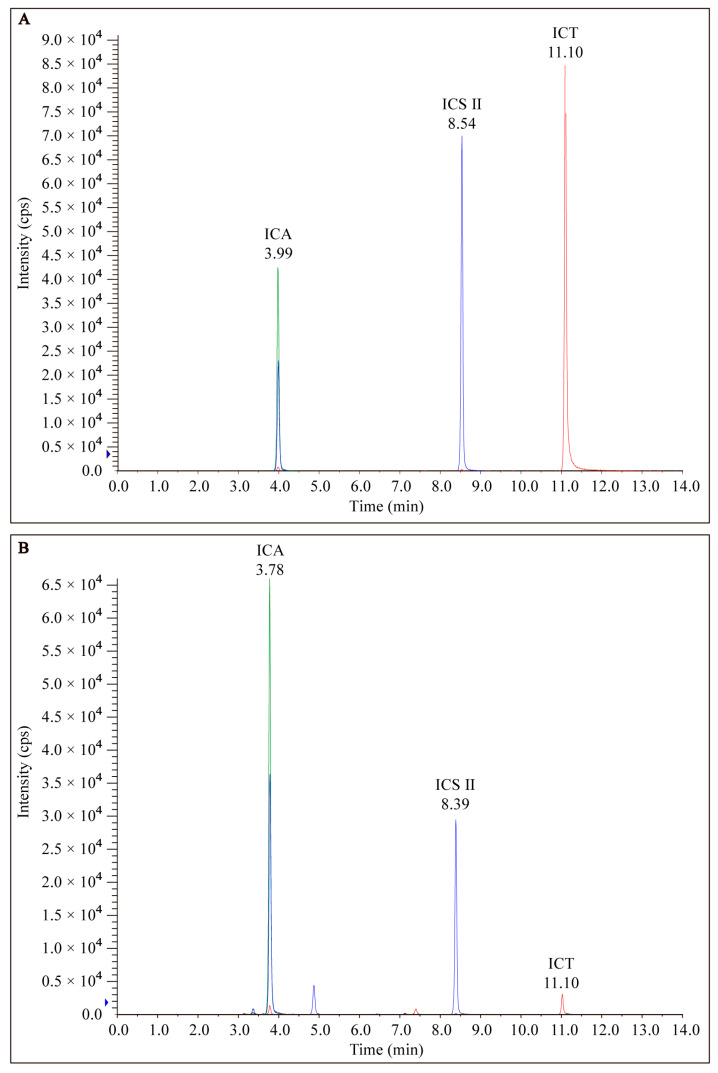
HPLC of the chromatogram of (**A**) standard marker compounds (ICA, ICS II, and ICT) found in EP and (**B**) EP.

EP was prepared from dried roots of *Epimedium* and analyzed via LC-MS/MS as described above. The chromatograms of EP from LC-MS/MS analyses exhibited several prominent peaks as well as many minor peaks (Figure 2). Among the peaks detected, both ICA, ICS II, and ICT were found to be the major constituents of EP. 

### 3.2. EP Exerts Neuroprotection on CIRI in Rats

**Figure 3:**
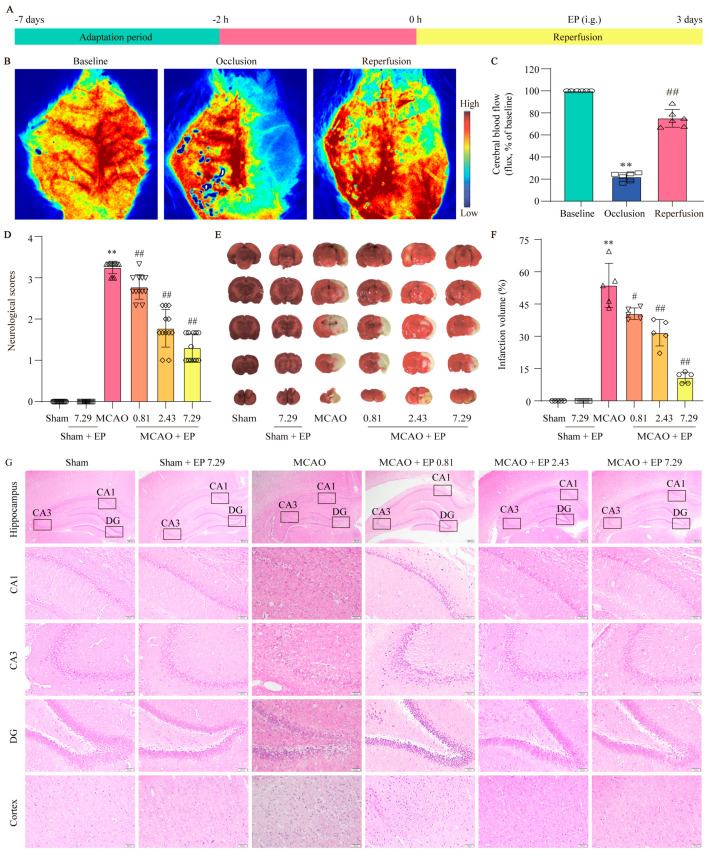
EP protects brain tissue against the damage induced by CIRI. Rats treated with EP (0.81, 2.43, and 7.29 g/kg) for 3 days had improved neurological function and a significant protective effect against brain damage caused by MCAO. (**A**) Schematic diagram of MCAO−induced rat model treated with EP. (**B**) Representative laser speckle images. (**C**) Quantitative analysis of rCBF, ** *p* < 0.01 versus baseline, ## *p* < 0.01 versus occlusion, *n* = 6. (**D**) Neurological function was assessed by a five-point scale, *n* = 12. (**E**,**F**) Representative images of TTC staining and quantification of infarct volumes 3 days after MCAO, *n* = 5. (**G**) H&E staining, *n* = 5 (×40, ×200; scale bar: 200 μm and 50 μm). Data presented are means ± SD. ** *p* < 0.01 versus sham group. # *p* < 0.05, ## *p* < 0.01 versus MCAO group.

To evaluate the protection of EP on CIRI, infarct volume and Longa 5 neurological deficient scores were determined, and H&E staining was done. We first assessed whether the MCAO model was successfully constructed by the LSCI system to detect rCBF. The results showed that rCBF decreased to 22 ± 4% of the baseline and lasted for 2 h after the middle cerebral artery of the rats was blocked via silicone-coated nylon monofilament. Removal of the nylon monofilament resulted in rapid reperfusion of rCBF to more than 80% of the baseline, indicating that the MCAO model was acceptable for the following experiments (Figure 3B,C). In addition, EP significantly decreased the neurological scores (Figure 3D) and infarct volume of rats compared with the MCAO group (Figure 3E,F). Furthermore, the results showed that neurons in the hippocampus and cortex disappeared or lacked a visible cell boundary after the MCAO insult, whereas EP reversed these changes in the hippocampus and cortex (Figure 3G). These findings suggest that EP effectively evokes neuroprotection on CIRI.

### 3.3. RNA-Seq Analysis of EA-Mediated Neuroprotection on CIRI

**Figure 4:**
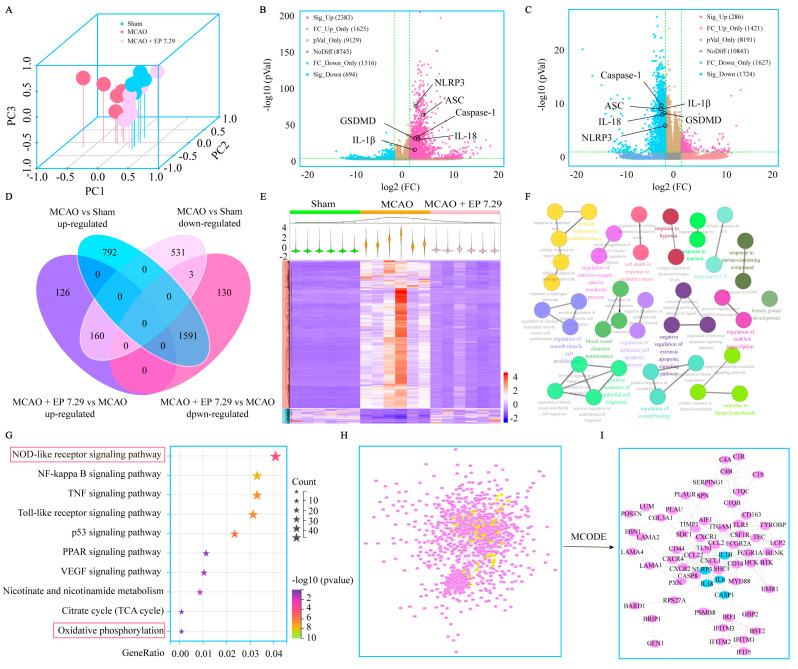
The global gene expression of EA−mediated protection against CIRI by RNA-Seq analysis. (**A**) PCA. (**B**) The fold change of DEGs of the MCAO group versus the sham group was summed up using a volcano plot. (**C**) The fold change of DEGs of the MCAO + EP 7.29 group versus the MCAO group was summed up using a volcano plot. (**D**) Venn, and (**E**) Hierarchical heatmap. (**F**) GO enrichment analysis of DEGs. (**G**) KEGG pathway analysis of DEGs. (**H**) The PPI network of DEGs. (**I**) Core targets in the PPI network.

DEGs were determined under the condition of both FC > 3 and *p* value < 0.05. There were 2383 significant up and 694 significant down DEGs between the MCAO group versus the sham group, and 286 significant up and 1724 significant down DEGs between the MCAO + EP 7.29 group versus the MCAO group (Figure 4B,C). The DEGs used for subsequent analysis in this study were up-regulated in the MCAO group versus the sham group and down-regulated in the MCAO + EP 7.29 group versus the MCAO group, or intersection genes down-regulated in the MCAO group versus the sham group and up-regulated in the MCAO + EP 7.29 group versus the MCAO group. Additionally, 1751 overlapped DEGs genes were observed between the two treatments of the MCAO group versus the sham group and the MCAO + EP 7.29 group versus the MCAO group (Figure 4D). Moreover, hierarchical clustering analysis showed that the expression profiles of the DEGs in the sham group and the MCAO + EP 7.29 group were significantly different from that of the MCAO group (Figure 4E). Furthermore, KEGG pathway analysis and enrichment of GO terms ascertained a considerable number of genes involved in the NOD-like receptor signaling pathway, oxidative phosphorylation (Figure 4F,G). In addition, the main targets (e.g., Nlrp3, Caspase-1, and Il-18) of the PPI network were found by the Cytoscape plugin MCODE (Figure 4I). These findings indicate that the neuroprotection EP evoked on CIRI was at least partially mediated through an NLRP3/pyroptosis signaling pathway.

### 3.4. The Main Components of EP Bind Directly to NLRP3

**Figure 5:**
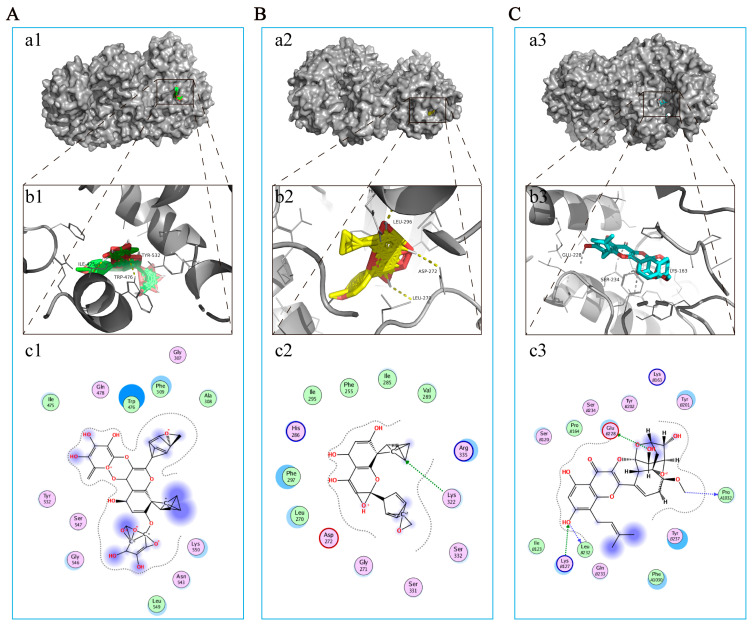
ICA, ICT, and ICS II directly bound to NLRP3. (**A**) ICA bound to NLRP3. (**a1**) Two-dimensional interaction map for ICA onto NLRP3 protein binding site. (**b1**) The entire view of the binding sites between ICA and NLRP3. (**c1**) A close-up view of the molecular binding pocket. (**B**) ICT bound to NLRP3. (**a2**) Two-dimensional interaction map for ICT onto NLRP3 protein binding site. (**b2**) The entire view of the binding sites between ICT and NLRP3. (**c2**) A close-up view of the molecular binding pocket. (**C**) ICS II bound to NLRP3. (**a3**) Two-dimensional interaction map for ICS II onto NLRP3 protein binding site. (**b3**) The entire view of the binding sites between ICS II and NLRP3. (**c3**) A close-up view of the molecular binding pocket.

As mentioned earlier, NLRP3 has been considered a potential therapeutic target for EP in CIRI. Correspondingly, molecular docking was used to further explore the interaction between the major compounds (ICA, ICT, and ICS II) and NLRP3. The results showed that the binding energies of ICA, ICT, and ICS II with NLRP3 were −3.68, −5.13, and −6.33 kcal/mol, respectively (Figure 5). The results suggest that ICA, ICT, and ICS II interact directly with NLRP3 through various binding sites and inhibit the NLRP3 signaling pathway.

### 3.5. EP Reduces NLRP3 Expression, Neuron Loss, and Neuronal Death in the Cortex and Hippocampus after CIRI

**Figure 6:**
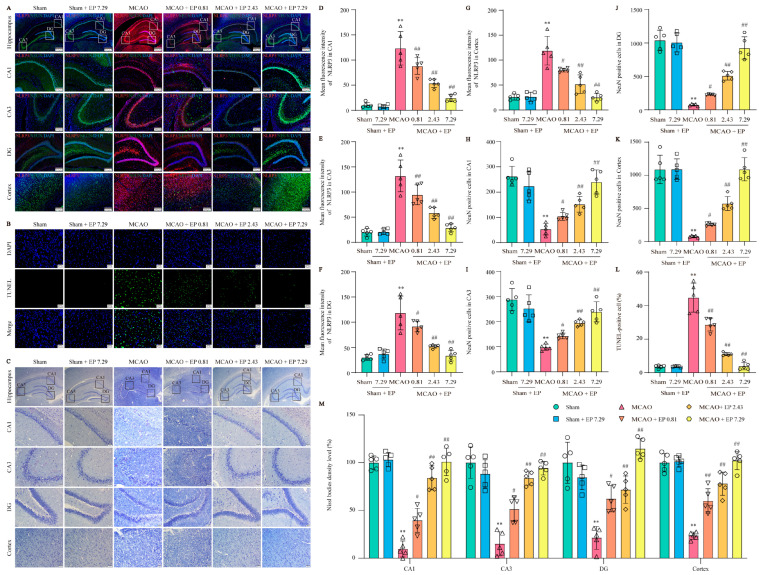
EP protects against CIRI-induced neuronal loss and neuronal death by reducing NLRP3 expression. (**A**) Representative images of NLRP3 (green) and NeuN (red) expression in the hippocampus and cortex (×40, ×100; scale bar: 500 μm and 200 μm). (**B**) Representative images of TUNEL staining in the cerebral cortex (×400; scale bar: 50 μm). (**C**) Nissl staining (×40, ×200; scale bar: 200 μm and 50 μm). (**D**–**G**) NLRP3 intensity in the hippocampal CA1, CA3, DG, and cortex regions, *n* = 5. (**H**–**K**) NeuN of cerebral in the hippocampal CA1, CA3, DG, and cortex regions, *n* = 5. (**L**) Quantitation of TUNEL positive cells, *n* = 5. (**M**) Nissl bodies density level (fold of sham) in the cortex and hippocampal CA1, CA3, and DG regions, *n* = 5. Data presented are means ± SD. ** *p* < 0.01 versus sham group. # *p* < 0.05, ## *p* < 0.01 versus MCAO group.

To further confirm the network analysis results, the expression of NLRP3 was determined using an IF experiment in the cortex and hippocampus (Figure 6A). Consistent with the results from the RNA-Seq analysis, CIRI caused a sharp increase in NLRP3 expression, whereas it was significantly reduced by EP treatment (Figure 6D–G). Additionally, CIRI induced neuronal loss in the cortex and hippocampus, whereas the number of neurons was significantly increased by EP administration (Figure 6H–K). Furthermore, the results showed that apoptotic cell number was increased in the MCAO group compared to those of the control group, whereas EP significantly reduced the neuronal death number in the cerebral cortex (Figure 6B,L). In addition, the results showed that CIRI induced a loss of Nissl bodies and shrunken nuclei neuronal atrophy in the cortex and hippocampus. However, EP markedly reversed this injury following CIRI (Figure 6G,M). These findings suggest that EP effectively protects against neuronal damage via inhibition of NLRP3.

### 3.6. EP Prevents CIRI-Induced Neuroinflammation through Down-Regulated NLRP3 and Its Downstream Pyroptosis-Related Genes

**Figure 7:**
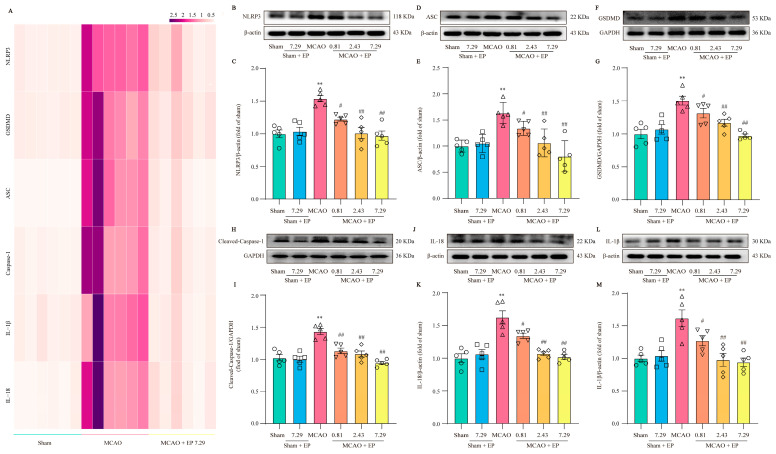
EP down-regulates NLRP3 and its downstream pyroptosis-related genes after CIRI insult. (**A**) Heatmap of NLRP3 and its downstream pyroptosis-related genes, *n* = 6. (**B**) Representative western blots of NLRP3. (**C**) Quantitation of NLRP3, *n* = 5. (**D**) Representative western blots of ASC. (**E**) Quantitation of ASC, *n* = 5. (**F**) Representative western blots of GSDMD. (**G**) Quantitation of GSDMD, *n* = 5. (**H**) Representative western blots of Cleaved-Caspase-1. (**I**) Quantitation of Cleaved-Caspase 1, *n* = 5. (**J**) Representative western blots of IL-18. (**K**) Quantitation of IL-18, *n* = 5. (**L**) Representative western blots of IL-1β. (**M**) Quantitation of IL-1β, *n* = 5. Data presented are means ± SEM. ** *p* < 0.01 versus sham group. # *p* < 0.05, ## *p* < 0.01 versus MCAO group.

RNA-Seq transcriptome expression analysis found that NLRP3 and pyroptosis-related genes were significantly up-regulated in the MCAO group, but these changes were significantly down-regulated after EP administration (Figure 7A). Furthermore, the expressions of the crucial proteins in NLRP3 and its downstream pyroptosis-related genes were determined using Western blot. The results showed that NLRP3, Cleaved-Caspase 1, ASC, GSDMD, IL-18, as well as IL-1β protein expressions, were observably increased in the ischemic penumbra of rats after CIRI compared to those of the sham group; however, EP significantly reversed these changes (Figure 7B–M). These findings indicate that the protective effect of EP on CIRI, at least partly, is due to the mediation of the NLRP3 signaling pathway.

### 3.7. EP Decreases the Activation of Microglia and Astrocytes, along with Reducing Proinflammatory Cytokines and Oxidative Stress Mediators

**Figure 8:**
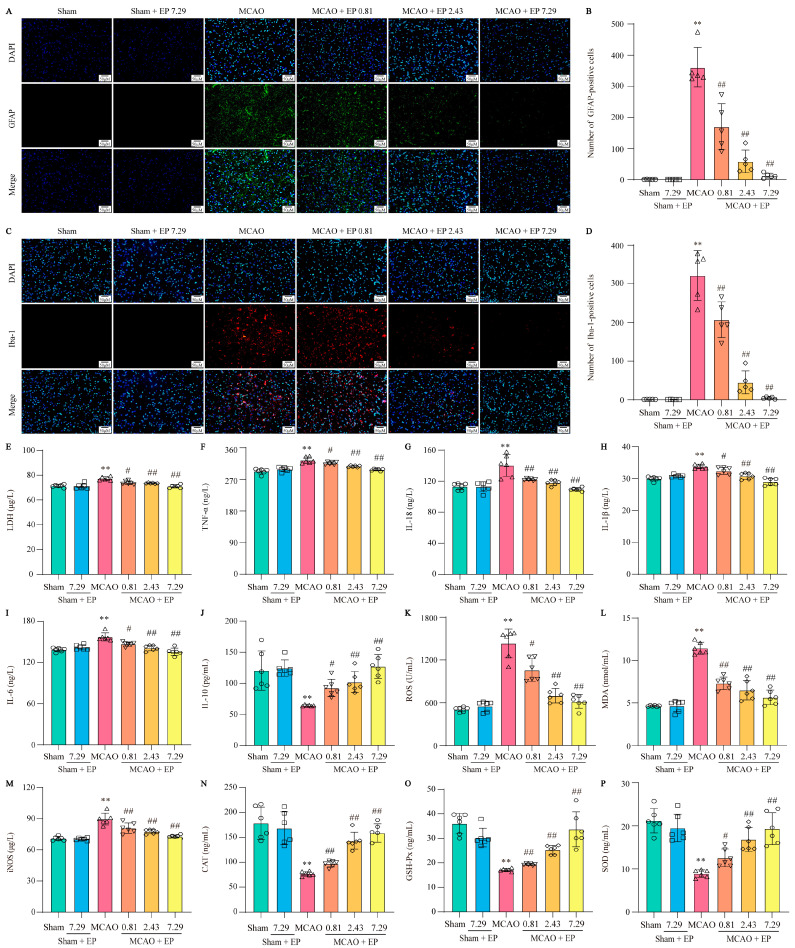
EP decreases the activation of astrocytes and microglia and inhibits neuroinflammation and oxidative stress. (**A**) Representative images of GFAP expression in cerebral cortex. (**B**) GFAP-positive cell numbers in cerebral cortex, *n* = 5. (**C**) Representative images of Iba-1 expression in cerebral cortex. (**D**) Iba-1-positive cell numbers in cerebral cortex, *n* = 5. (**E**) LDH level, *n* = 6. (**F**) TNF-α level, *n* = 6. (**G**) IL-18 level, *n* = 6. (**H**) IL-1β level, *n* = 6. (**I**) IL-6 level, *n*= 6. (**J**) IL-10 level, *n* = 6. (**K**) ROS level, *n* = 6. (**L**) MDA level, *n* = 6. (**M**) iNOS level, *n* = 6. (**N**) CAT activity, *n* = 6. (**O**) GSH-Px activity, *n* = 6. (**P**) SOD activity, *n* = 6. Data were shown as mean ± SD. ** *p* < 0.01 versus sham group. # *p* < 0.05, ## *p* < 0.01 versus MCAO group.

EP treatment significantly inhibited the activation of astrocytes and microglia and suppressed the expression levels of inflammatory cytokines, showing strong anti-inflammatory activity. Astrocytes and microglia are critical cells in the development of neuro-inflammation, so specific markers GFAP and Iba-1 were used to label activated astrocytes and microglia in the cerebral cortex, respectively. CIRI in rats resulted in massive astrocyte and microglial activation, which was significantly reversed after three days of EP treatment (Figure 8A–D). Additionally, an LDH test measured the level of LDH in blood to check for tissue damage. EP effectively decreased the level of LDH in a concentration-dependent manner (Figure 8E). CIRI caused a sharp increase of multiple inflammatory cytokines, such as TNF-α, IL-18, IL-1β, and IL-6. In contrast, EP treatment obviously decreased these inflammatory cytokines (Figure 8F–I). Notably, EP treatment not only reduced inflammatory cytokines but also increased levels of the anti-inflammatory factor IL-10 in rats with CIRI (Figure 8J). OS, like inflammation, is a major pathological process that accompanies CIRI in the brain. Based on the results of the previous GO analysis, ELISA assays were used to measure OS mediators. In the rat model of CIRI, cellular and mitochondrial ROS, MDA, and iNOS levels were increased, and CAT, GSH-Px, and SOD activity were decreased compared to the control. However, these changes were reversed by EP in a dose-dependent manner after the CIRI insult (Figure 8K–P). These findings suggest that EP can effectively inhibit the occurrence of CIRI-induced inflammation and oxidative stress.

## 4. Discussion

**Figure 9:**
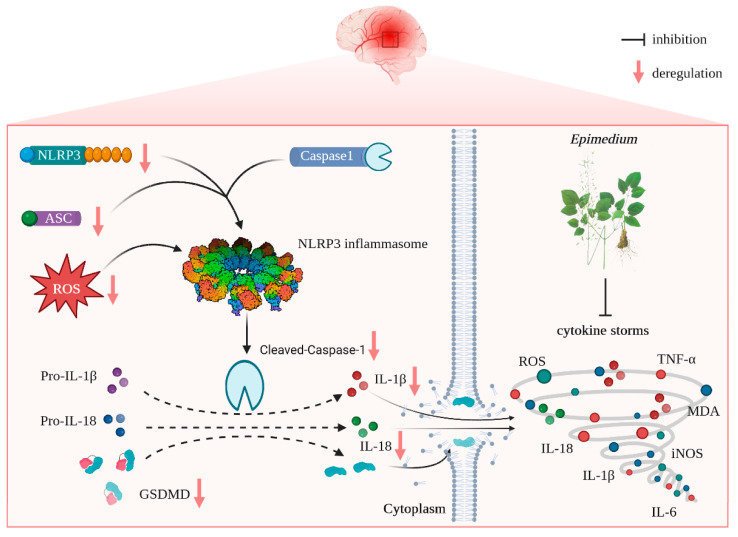
Schematic illustration of molecular mechanisms for the protective effect of EP against CIRI. EP evokes robust neuroprotection on CIRI via targeting NLRP3 through ROS-mediated pyroptosis.

The present study reveals that (i) EP aqueous extract rescues CIRI, along with reducing neuron loss due to its anti-neuroinflammation and antioxidant properties, and (ii) the neuroprotective effects of EP on CIRI by targeting NLRP3 through ROS-mediated pyroptosis signaling pathway (Figure 9). Corporately, our findings unveil the potential target of EP against CIRI and come up with a “proof-of-concept” for EP to conquer CIRI.

To date, the desired curative options on CIRI are unavailable in clinics due to their intricate pathogenesis. Emerging evidence suggests that neuroinflammation and oxidative stress play crucial roles in exacerbating neurological dysfunction and neuron loss after CIRI [18]. Therefore, efficacious agents to conquer CIRI should own potent anti-neuroinflammation and antioxidant activities. Previous research has found that the main active compounds of EA, namely ICA, ICS II, and ICT, can effectively penetrate the blood-brain barrier and demonstrate potent anti-neuroinflammatory and antioxidant activities against multiple diseases [10,12,19,20]. The above-mentioned scenario and pharmacological properties of EP prompted us to excavate the therapeutic effect and its possible targets of EP on CIRI.

Our findings demonstrated that EP effectively attenuated the neurological function deficit and the cerebral infarct volume, as evidenced by behavioral and neurological assessment and TTC staining, which suggested that EP could protect against CIRI. However, the comprehensive, detailed mechanism of EP is still unknown. 

We thereafter predicted the possible underlying mechanism of EP on CIRI using RNA-Seq analysis and molecular docking simulations. The results foretold that oxidative stress- and pyroptosis-related pathways were involved in the beneficial effect of EP following CIRI, and Nlrp3 was identified as the dominant DEG. Interestingly, ICA, ICS II, and ICT, the major active compounds of EA, could directly bind to NLRP3. These findings suggest that EP might intervene in NLRP3-mediated CIRI pathological conditions. 

Mounting evidence demonstrates that NLRP3 is activated within hours of the onset of IS and drives neuroinflammation, eventually leading to neuronal death [21]. As we expected, EP significantly reduced NLRP3 expression, neuron loss, and neuronal death in the cortex and hippocampus of the ischemic penumbra, as reflected by IF staining, Nissl staining, and TUNEL staining. Furthermore, EP also down-regulated the protein expression of NLRP3 and its downstream pyroptosis-related genes after CIRI insult, in keeping with the data of the RNA-seq analysis. These findings reveal that EP could rescue neuronal damage in the ischemic penumbra dependent on suppressing the activation of NLRP3-mediated pyroptosis. Moreover, microglia and astrocytes are activated upon stimulation by NLRP3, thereby promoting proinflammatory cytokines release and oxidative stress and ultimately resulting in neuronal loss. We next observed the effect of EP on neurogliocyte activation. Our results showed that EP markedly decreased the activation of microglial cells and astrocytes, along with reducing proinflammatory cytokines and oxidative stress mediators. These findings suggest that the protective effect of EP is due to its anti-neuroinflammation and antioxidant properties.

Notwithstanding the encouraging findings, there are still limitations in the present study. First of all, although we discovered that EP displays a beneficial effect on CIRI, there is unavailable direct evidence to elucidate how the *Epimedium*, which is administered by oral gavage, may reach the brain after a stroke and whether EP can protect against the blood-brain barrier challenged CIRI; additionally, its metabolic processes in the brain are still ill-defined. Thus, more compelling evidence should be offered to investigate the effect of EP on the long-term neurological functional recovery of EP following CIRI. Moreover, long-term toxicity tests and, ultimately, large and well-designed cohort clinical trials of EP also should be provided in the future.

In summary, our findings discovered for the first time that EP evokes robust neuroprotection on CIRI via targeting NLRP3 through ROS-mediated pyroptosis. Thus, it is concluded that EP may be a promising weapon to combat IS.

## Data Availability

All data related to this research are presented in the manuscript.
